# Genetic polymorphisms in *TNIP1* increase the risk of gastric carcinoma

**DOI:** 10.18632/oncotarget.9637

**Published:** 2016-05-26

**Authors:** Zhao Liu, Yuting Shi, Yuyan Na, Qi Zhang, Sizhe Cao, Xianglong Duan, Xiyang Zhang, Hua Yang, Tianbo Jin, Yiming Li

**Affiliations:** ^1^ Department of General Surgery, The Second Affiliated Hospital, Xi'an Jiaotong University, Xi'an 710004, China; ^2^ Department of Surgery, Xi'an Chest Hospital, Xi'an TB&Thoracic Tumor Hospital, Xi'an 710100, China; ^3^ Department of Medical Oncology, Graduate School of Inner Mongolia Medical University, Hohhot 010000, China; ^4^ Department of Pediatric Orthopedics, The Second Affiliated Hospital of Inner Mongolia Medical University, Hohhot 010030, China; ^5^ Department of Medical, Xi'an Chest Hospital, Xi'an TB&Thoracic Tumor Hospital, Xi'an 710100, China; ^6^ Second Department of General Surgery, Shaanxi Province People's Hospital, Xi'an 710001, China; ^7^ Department of Biochemistry, School of Life Sciences, Northwest University, Xi'an 710069, China

**Keywords:** gastric carcinoma, single nucleotide polymorphisms (SNPs), TNF-α-induced protein 3-interacting protein 1 (TNIP1), case-control study

## Abstract

The distribution and levels of *TNIP1* in malignant and normal gastric mucosa are different, but it is not known whether *TNIP1* polymorphisms are related to gastric carcinogenesis. To assess the association between four *TNIP1* SNPs (rs3792792, rs4958881, rs7708392, rs10036748) and carcinogenesis, we used Sequenom Mass-ARRAY technology to determine the genotypes of 302 gastric carcinoma patients and 300 healthy controls in a Northwest Chinese Han population. These data were then compared using the Chi-square test/Fisher's exact test, genetic model analysis, and haplotype analysis. Odds ratios (OR) and 95% confidence intervals (CI) were used to evaluate the correlation. We observed that patients with the “G” allele of rs7708392 and the “C” allele of rs10036748 showed an increased risk of gastric carcinoma (OR= 1.335, 95%CI: 1.021-1.745, *P*= 0.035; OR= 1.358, 95%CI: 1.039-1.774, *P*= 0.025, respectively). Conversely, the haplotype “CT” of *TNIP1* (rs7708392-rs10036748) may act as a genetic protective factor for gastric carcinoma (adjusted OR= 0.731, 95%CI: 0.552-0.970, *P*= 0.030). Our results are the first to suggest that genetic variation in *TNIP1* gene is associated with gastric carcinoma, though, this finding must be confirmed in other populations with larger sample size.

## INTRODUCTION

About one million new cases of gastric carcinoma (GC) were estimated to have occurred in 2008, making it the fourth most common malignant tumor worldwide. GC was the second leading cause of cancer-related death 738,000 deaths in the world. The incidence of GC was highest in Eastern Asia and the highest mortality rate was observed in Eastern Asia, specifically in China [1]. Since symptoms of early stage GC are not typical, patients usually diagnosed in the advanced stage after the optimal time for therapy. Although surgery, chemotherapy, and radiotherapy have improved the survival of early stage patients [2], the therapy and prognosis of advanced patients are still poor [3]. Given the lethality of GC on survival, identification of risk factors for oncogenesis and new strategies for primary prevention are necessary.

The pathogenesis of GC is not completely clear. GC is a complex and heterogeneous disease influenced by genetic and environmental factors [4]. Environmental factors including dietary habits, smoking and chronic atrophic gastritis caused mainly by Helicobacter pylori infections are known GC risk factors [5]. However, not all people exposed to these hazards eventually suffer from GC; for example, only 3% people infected with H. pylori develop GC [6, 7]. Hence, genetic susceptibility may play a more important role in gastric carcinogenesis.

TNF-α-induced protein 3-interacting protein 1 (*TNIP1*) on chromosome 5q33, also known as VAN, NAF1, ABIN-1, and nip40-1, encodes an A20-binding protein which plays an important role in autoimmunity, chronic inflammation, and cancer through the inhibition of nuclear factor kappa-B (NF-κB) activation [8]. Epidermal growth factor receptor (EGFR) induced activation of the transcription factor NF-κB may be involved in the malignant behavior of EGFR overexpressing tumor cells, such as in lung cancer and gastric carcinoma [9, 10]. L Huang et al. believed that EGF-induced NF-κB activation could be inhibited by overexpression of ABINs, including ABIN-1. Knockdown of ABIN-1 by RNA interference boosted the NF-κB response to EGF stimulation [11]. We propose that *TNIP1* might be a protective gene involved in the inhibition of oncogenesis.

In several malignant tissues, *TNIP1* staining is often altered from its distribution and levels in normal tissues, such as in gastric lining, glandular epithelia with moderate cytoplasmic and weak nuclear staining, while far less staining tended to be associated with cell periphery. Igor Gurevich et al. thought the altered distribution and levels of *TNIP1* in malignant tissues may affect processes in which *TNIP1* is involved, such as NF-κB signaling, possibly contributing to malignant tumor development [8]. We set out to determine whether *TNIP1* is related to gastric carcinogenesis.

The *TNIP1* gene has been implicated in susceptibility to a number of autoimmune diseases, such as systemic lupus erythematosus (SLE), systemic sclerosis and rheumatoid arthritis (RA). Single nucleotide polymorphisms in the *HLA-DRB1* [12–14] and *IL23R* genes [15, 16] can alter susceptibility to GC and autoimmune diseases, such as SLE and systemic sclerosis. But, whether *TNIP1* gene is also the shared risk gene for GC and autoimmune diseases is unknown.

To investigate the association between *TNIP1* and GC risk, we genotyped 4 variants associated with SLE and systemic sclerosis [17–19], rs3792792, rs4958881, rs7708392, rs10036748, and analyzed the difference between GC patients and matched controls from the Chinese Han population from Northwest China.

## RESULTS

302 GC patients and 300 healthy controls were enrolled in our study. We show that age (*P*<0.001) and gender (*P*<0.001) were significantly different between GC cases and health controls in Table 1. In order to eliminate those residual confounding effects, the variable of age and gender were adjusted in multivariate unconditional logistic regression analysis.

**Table 1:** Characteristics of cases and controls in this study

Variable	Cases (n=302)	Controls (n=300)	*P* value
Sex			<0.001
Male	233 (77.2%)	180 (60.0%)	
Female	69 (22.8%)	120 (40.0%)	
Age, yr	58.01	60.42	<0.001

*P* values were calculated from pearson Chi-square test.

The candidate *TNIP1* gene SNPs (rs3792792, rs4958881, rs7708392, and rs10036748) were genotyped in GC patients and healthy controls. One SNP (rs4958881) was excluded due to significant deviation from Hardy-Weinberg equilibrium (*P*<0.05); the remaining three SNPs were in accordance with the Hardy-Weinberg equilibrium in the control group with a value of *P*>0.05. We compared the differences in frequency distributions of alleles between GC cases and controls by Chi-square test/Fisher's exact test and found two significant SNPs in the *TNIP1* gene were associated with GC risk (Table 2). The frequency of the “G” allele of rs7708392 and the “C” allele of rs10036748 were significantly higher in GC cases than in controls (26.0% versus 20.8%; 26.3% versus 20.8%, respectively). And the “G” allele of rs7708392 and the “C” allele of rs10036748 showed significantly increased risk of GC (OR= 1.335, 95%CI: 1.021-1.745, *P*= 0.035; OR= 1.358, 95%CI: 1.039-1.774, *P*= 0.025, respectively). The frequencies of heterozygous variants “GC” genotype in rs7708392 and “CT” genotype in rs10036748 significantly differed in GC cases and controls (Table 3). After further adjustment by age and gender, the difference of “GC” genotype in rs7708392 and “CT” genotype in rs10036748 remains significant (adjusted OR= 1.433, 95%CI: 1.013-2.029, *P*= 0.042; adjusted OR= 1.446, 95%CI: 1.021-2.048, *P*= 0.038, respectively)

**Table 2:** Allele frequencies in cases and controls and odds ratio estimates for gastric carcinoma

SNP	Chromosome	Position	Allele	Minor allele frequency		HWE *P* value	OR (95%CI)	*P*^a^
				Case	Control			
rs3792792	5	150440506	C/T	0.076	0.062	1.0000	1.254 (0.801-1.964)	0.321
rs4958881	5	150450236	C/T	0.119	0.114	3.30E-45	1.056 (0.739-1.511)	0.763
rs7708392	5	150457485	G/C	0.260	0.208	0.8612	1.335 (1.021-1.745)	0.035^*^
rs10036748	5	150458146	C/T	0.263	0.208	0.8612	1.358 (1.039-1.774)	0.025^*^

HWE: Hardy-Weinberg equilibrium; OR: odds ratio; 95%CI: 95% confidence interval.

a *P* values were calculated from Chi-square test/Fisher's exact test.

* *P*≤0.05 indicates statistical significance.

**Table 3:** Genotypes of the four SNPs and their associations with risk of gastric carcinoma

SNP	Genotype	Genotype frequency		Without adjustment		With adjustment	
		Cases(N)	Controls(N)	OR (95%CI)	*P^a^*	OR (95%CI)	*P^b^*
rs3792792	TT	261	264	1.000	-	1.000	-
	CT	36	35	1.040 (0.634-1.708)	0.876	1.116 (0.668-1.865)	0.674
	CC	5	1	5.057 (0.587-43.580)	0.140	5.766 (0.621-53.580)	0.123
rs4958881	TT	258	257	-	-	-	-
	CT	0	0	-	-	-	-
	CC	35	33	-	-	-	-
rs7708392	CC	162	187	1.000	-	1.000	-
	GC	123	101	1.406 (1.004-1.969)	0.048^*^	1.433 (1.013-2.029)	0.042^*^
	GG	17	12	1.635 (0.758-3.526)	0.210	1.518 (0.689-3.344)	0.300
rs10036748	TT	161	187	1.000	-	1.000	-
	CT	123	101	1.414 (1.010-1.982)	0.044^*^	1.446 (1.021-2.048)	0.038^*^
	CC	18	12	1.742 (0.815-3.726)	0.152	1.606 (0.736-3.506)	0.235

SNP: Single nucleotide polymorphism; OR: odds ratio; 95%CI: 95% confidence interval.

a *P* values were calculated from unconditional logistic regression analysis.

b *P* values were calculated by unconditional logistic regression analysis with adjustments for age and gender.

* *P*≤0.05 indicates statistical significance.

Next, we assumed that the minor allele of each SNP was a risk factor and analyzed the association between each variant and GC under three genetic models (Table 4). Two susceptibility SNPs were found to be associated with increased risk of GC both before and after the adjustment: rs7708392 under the dominant model (adjusted OR= 1.443, 95%CI: 1.032-2.017, *P*= 0.032) and under the additive model (adjusted OR= 1.346, 95%CI: 1.013-1.786, *P*= 0.040) and rs10036748 under the dominant model (adjusted OR= 1.464, 95%CI: 1.047-2.047, *P*= 0.026) and under the additive model (adjusted OR= 1.367, 95%CI: 1.031-1.813, *P*= 0.030).

**Table 4:** Association between SNPs and gastric carcinoma in multiple inheritance models

SNP	Model	Genotype	Without adjustment		With adjustment	
			OR (95%CI)	*P*^a^	OR (95%CI)	*P*^b^
rs3792792	Dominant	T/T	1		1	
		C/C+C/T	1.152 (0.713-1.860)	0.563	1.236 (0.752-2.032)	0.404
	Recessive	C/T+T/T	1		1	
		C/C	5.034 (0.585-43.340)	0.141	5.687 (0.613-52.760)	0.126
	Additive	-	1.234 (0.801-1.900)	0.341	1.315 (0.837-2.064)	0.235
rs4958881	Dominant	T/T	1		1	
		C/C+C/T	1.056 (0.637-1.752)	0.831	1.129 (0.668-1.908)	0.652
	Recessive	C/T+T/T	1		1	
		C/C	1.056 (0.637-1.752)	0.831	1.129 (0.668-1.908)	0.652
	Additive	-	1.028 (0.798-1.324)	0.831	1.062 (0.817-1.381)	0.652
rs7708392	Dominant	C/C	1		1	
		G/G+G/C	1.430 (1.033-1.980)	0.031^*^	1.443 (1.032-2.017)	0.032^*^
	Recessive	G/C+C/C	1		1	
		G/G	1.432 (0.672-3.052)	0.353	1.319 (0.606-2.871)	0.485
	Additive	-	1.351 (1.027-1.778)	0.032^*^	1.346 (1.013-1.786)	0.040^*^
rs10036748	Dominant	T/T	1		1	
		C/C+C/T	1.449 (1.047-2.006)	0.025^*^	1.464 (1.047-2.047)	0.026^*^
	Recessive	C/T+T/T	1		1	
		C/C	1.521 (0.720-3.216)	0.272	1.390 (0.645-2.998)	0.401
	Additive	-	1.373 (1.045-1.806)	0.023^*^	1.367 (1.031-1.813)	0.030^*^

SNP: Single nucleotide polymorphism; OR: odds ratio; 95%CI: 95% confidence interval.

a *P* values were calculated from unconditional logistic regression analysis.

b *P* values were calculated by unconditional logistic regression analysis with adjustments for age and gender.

* *P*≤0.05 indicates statistical significance.

**Figure 1:**
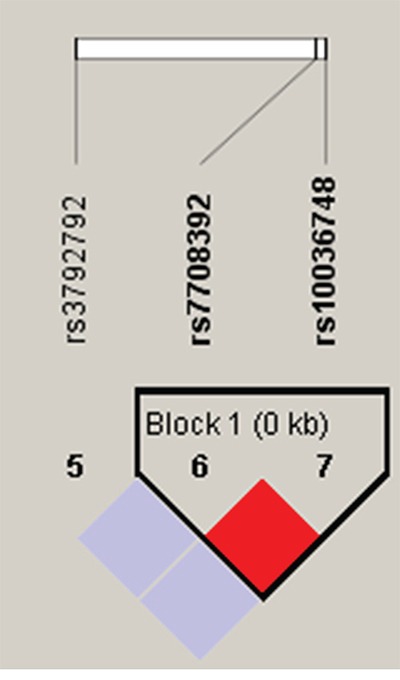
Haplotype block map for part of the SNPs in *TNIP1* gene Standard color frame is used to show LD pattern. Increasing color depth of red indicates increasing degree of LD, with dark red for very strong LD.

Finally, the haplotypes with frequencies of more than 0.05 were selected for further research (Table 5). In Figure 1, the red squares of the *TNIP1* linkage disequilibrium (LD) block exhibited statistically significant linkage between rs7708392 and rs10036748. We observed that the “CT” haplotype was more frequent among GC cases and may have a protective effect against GC both before and after the adjustment (adjusted OR= 0.731, 95%CI: 0.552-0.970, *P*= 0.030).

**Table 5:** *TNIP1* haplotype frequencies and the association with gastric carcinoma risk

Haplotype block	Haplotype frequencies		Without adjustment		With adjustment	
	Case	Control	OR (95%CI)	*P^a^*	OR (95%CI)	*P^b^*
CT	0.737	0.792	0.728 (0.554-0.957)	0.023^*^	0.731 (0.552-0.970)	0.030^*^
GCA	0.331	0.328	1.014 (0.791-1.299)	0.915	1.011 (0.783-1.307)	0.931

OR: odds ratio; 95%CI: 95% confidence interval.

a *P* values were calculated from unconditional logistic regression analysis.

b *P* values were calculated by unconditional logistic regression analysis with adjustments for age and gender.

* *P*≤0.05 indicates statistical significance.

## DISCUSSION

The present case-control study of 302 GC patients and 300 healthy controls was designed to investigate whether the four variants within the *TNIP1* gene are related to the risk of developing GC. We found that rs7708392 and rs10036748 in the *TNIP1* gene were significantly associated with GC risk in the Chinese Han population of Northwest China. The “G” allele of rs7708392 and the “C” allele of rs10036748 were identified as risk alleles for the development of GC. We also found that a haplotype “CT” of *TNIP1* gene was associated with a 27% reduction in the risk of GC.

However, it was surprising that *TNIP1* heterozygotes (GC for rs7708392 and CT for rs10036748) rather than homozygotes were significantly associated with GC risk. The phenomenon may be explained by the co-dominant heredity in which each of the two different alleles has its own effects on the specific protein synthesis and function. This assumption should be tested in future gene functional experiments.

NF-κB is constitutively activated in GC and activated or deregulated NF-κB is related to several aspects of oncogenesis, including promoting tumor cell proliferation, preventing apoptosis, and increasing tumor angiogenesis potentials [20, 21]. Nevertheless NF-κB activity is tightly controlled by several regulatory proteins, such as *TNIP1* (ABIN-1) which can inhibit the NF-κB activation induced by tumor necrosis factor, interleukin-1, EGF and lipopolysaccharide [11, 22]. We regarded *TNIP1* as a “protective” gene that may be involved in the inhibition of GC development. It is possible that polymorphisms that down-regulate expression of *TNIP1* gene render individuals susceptible to GC. This speculation is supported by our results that the “G” allele of rs7708392 and the “C” allele of rs10036748 were potential risk factors for gastric carcinogenesis.

Aya Kawasaki et al. found that rs7708392 was an risk factor for SLE in a Japanese population. Other studies observed that SNPs rs7708392 and rs10036748 in *TNIP1* are in strong linkage disequilibrium with SLE. As Sahil Gambhir and colleagues described, inflammation and gastrointestinal cancers can be connected by a critical mechanism of the NF-κB pathway [23]. Chronic infections and autoimmune processes give rise to prolonged specific inflammation which induces constitutive NF-κB activity, increasing the probability of developing specific cancers through downstream proteins. As a result, we conclude that polymorphisms in the *TNIP1* gene increase the possibility of developing neoplasms.

Several limitations in our present case-control study should be pointed out. First, the small sample size cannot provide sufficient statistical power to reflect the real association between SNPs in the *TNIP1* gene and GC. Second, the associations between polymorphisms in the *TNIP1* gene and histological subtype of GC were not discussed. Third, Helicobacter pylori infection and dietary habits are crucial factors in risk of gastric carcinogenesis, which were not included due to lack of corresponding clinical information. In addition, the associations we reported have not been investigated before; thus, further research with a larger sample size is needed to confirm our data.

Our present study provides evidence that single nucleotide polymorphisms in the *TNIP1* gene are associated with GC in the Chinese Han population from Northwest China. It is possible that these variants are GC risk factors and these data can provide a theoretical foundation for other researchers to further study the association between the *TNIP1* gene and GC risk in the Chinese Han or other populations.

## MATERIALS AND METHODS

### Study subjects

This study consisted of 302 GC patients and 300 healthy controls (Table 1). The cases were recruited at the Second Affiliated Hospital, Xi'an Jiaotong University and Shaanxi Province People's Hospital between March 2013 and June 2015. Inclusion and exclusion criterias were as follows: ➀ All subjects were ethnically homogeneous Chinese Han and residents of Northwest China. ② All included cases were recently diagnosed and histopathologically confirmed gastric cancer according to the World Health Organization (WHO) criteria [24]. ③ None of the GC patients had inflammatory, autoimmune disorders, and family history of cancer. ④ All patients who underwent radiotherapy and/or chemotherapy were excluded. All healthy controls had never been diagnosed with cancer and were interviewed by professional interviewers for their gender, age, and exposure to exogenous risk factors for malignant tumor such as smoking status, poor diet, occupational exposure to carcinogens, and family history of cancer. Those who possessed these exogenous risk factors were excluded from our study. All individuals involved in this study gave written informed consent for the genetic analysis. The study protocol was approved by the ethics committee of the Second Affiliated Hospital, Xi'an Jiaotong University.

### DNA isolation and genotyping

Blood samples were drawn from all subjects before they had received other therapies, such as surgery, radiotherapy, and chemotherapy. Genomic DNA was isolated from peripheral blood leukocytes in whole blood using the GoldMag-Mini Purification Kit (GoldMag Co. Ltd. Xi'an city, China) according to the manufacturer's instructions. DNA concentrations were measured using the NanoDrop 2000 (Thermo Scientific, Waltham, Massachusetts, USA) at wavelengths of A260 and A280 nm. DNA was quantified and diluted using QIAgility to a final concentration of 20ng/μl. Using the HapMap database, we searched SNPs from the *TNIP1* gene and restricted with a minor allele frequency (MAF) > 5% in the Chinese Han Beijing population. Four SNPs in the *TNIP1* gene were randomly selected for further genotyping. Primers for amplification process and single base extension reactions were designed with Sequenom Mass-ARRAY Assay Design 3.0 Software (Sequenom Co. Ltd, San Diego, California, USA) [25]. Subsequent SNP genotyping was performed using Sequenom Mass-ARRAY RS1000 (Sequenom, San Diego, CA). The corresponding primers used for each SNP in the present study are listed in Table 6. Data management and analysis were performed using Sequenom Typer 4.0 Software (Sequenom Co. Ltd) [25, 26].

**Table 6:** Summary of the primers used for the analysis of the TNIP1 polymorphisms

SNP	First PCRP (5′→3′)	Second PCRP (5′→3′)	UEP SEQ (5′→3′)
rs3792792	ACGTTGGATGCTC AGATCAGTTCACTCCTC	ACGTTGGATGATGG CAGCTGTTACGGCCAC	ccctTTACGGCCA CCACCAAGCATG
rs4958881	ACGTTGGATGCAC AAATATGTGGACAGTTT	ACGTTGGATGTGC AATTCCACCCAAGGATG	GGATGAAAGGAAGTGAGA
rs7708392	ACGTTGGATGAGG CCAACTGGTCAATTCTC	ACGTTGGATGGGG TCTCTTCTGGAACTTAG	ggggaTGGAACTTA GTAGACTAGTCA
rs10036748	ACGTTGGATGGCAAAGCAGCCCCTTTTTTC	ACGTTGGATGGCCAG TGGGAATGCAAAATG	tgtcATGCAAAA TGAAACAGACACTT

SNP, single-nucleotide ploymorphisms; PCRP, PCR primer; UEP, Un-extended mini-sequencing primer.

### Statistical analysis

All statistical analysis was conducted using Microsoft Excel and SPSS 16.0 (SPSS, Chicago IL USA). Allele frequency of each SNP in the control subjects was analyzed using the exact test to determine whether the four SNPs departed from Hardy-Weinberg equilibrium (HWE). We used Chi-square test/Fisher's exact test to compare the differences in SNP allele and genotype distribution between GC cases and controls [27]. Then the association between each SNP and GC was assessed under three genetic models: dominant, recessive and additive model using PLINK software, a web-based program available at http://pngu.mgh.harvard.edu/purcell/plink/. Finally, the SHEsis software platform (http://www.nhgg.org/analysis) and Haploview software package (version 4.2) were used to analyze and visualize patterns of linkage disequilibrium (LD) and haplotype construction [28]. The odd ratio (OR) and 95% confidence intervals (CI), calculated by using unconditional logistic regression analysis with adjustments for age and gender, were used to assess the association between each SNP and the risk of GC [29]. Two-sided *P*≤0.05 was considered statistically significant for all statistical tests.
